# Phenotypic markers misclassify isolates of *Salmonella* Typhimurium as *S.* Typhi, but were correctly identified by whole genome sequencing during a septicemia study in western Kenya

**DOI:** 10.1371/journal.pgph.0004773

**Published:** 2025-10-23

**Authors:** Joseph Kaingu, Kimita Gathii, Carolyne Kifude, James Nonoh, Lillian Ogonda, Amos Onditi, Kirti Tiwari, John Waitumbi

**Affiliations:** 1 Walter Reed Army Institute of Research-Africa/Kenya Medical Research Institute, Kisumu, Kenya; 2 Department of Biomedical Sciences, School of Public Health, Maseno University, Maseno, Kenya; University of Cape Town, SOUTH AFRICA

## Abstract

Antimicrobial resistance (AMR) is a rising global health threat and estimated to cause 700,000 deaths annually. Although blood cultures (BCs) are the reference standard to diagnose bloodstream infections and inference of antimicrobial susceptibility testing (AST), the method could fail to differentiate bacteria with similar biochemical characteristics. This study compared phenotypic method (AST) and whole genome sequencing (WGS) in bacterial species identification and assessment of AMR. Blood samples came from children who had been prescribed antibiotics at admission at the county referral hospitals in western Kenya and around the Lake Victoria region. BCs and AST were performed on BD Bactec 9050 and Phoenix 100 respectively. Out of the 960 BCs, 17 isolates were useable and were evaluated by WGS on the Oxford Nanopore PromethION platform. BD Phoenix system identified the 17 bacteria isolates as: 4 *Escherichia coli*, 8 *Salmonella enterica* serovar Typhi, 1 unspeciated *Salmonella*, 3 *Staphylococcus aureus* and 1 *Streptococcus pneumoniae*. WGS results differed from BCs in identifying the 9 *Salmonella* species, with WGS identifying the species as *Salmonella enterica* serovar Typhimurium. Conversely, WGS detected AMR determinants in bacteria that AST had classified as susceptible. In conclusion, we caution that BCs may not be providing correct identity of *Salmonella* species. The observed discrepancies between phenotypic and genotypic markers of drug resistance highlight the challenges in interpreting and predicting the functional utility of AMR determinants.

## Introduction

Blood cultures (BCs) serve as the gold standard for diagnosing bloodstream infections and when coupled with antimicrobial susceptibility testing (AST), provide the associated antimicrobial resistance (AMR) profiles. AMR is a major global public health challenge which threatens to render antibiotic management strategies ineffective [1]. Sub-Saharan Africa shoulder’s the highest morbidity and mortality burden, and the associated high AMR treatment costs [2,3]. The misuse of antibiotics in humans and animals is a major driver of AMR, and leads to poor infection control, environmental contamination, and geographical spread of resistant bacteria [4]. The identification of AMR determinants (genes and mutations) can supplement traditional phenotypic antimicrobial susceptibility testing (AST) by predicting bacterial resistance to specific antibiotics, helping to guide the selection of effective treatments [5].

Correct identification of bacteria species is important for proper attribution and for epidemiological tracking. Unfortunately, certain bacteria exhibit similar biochemical characteristics, making differentiation through traditional culture methods challenging. In such cases, molecular techniques like PCR-based identification and sequencing of the 16S rRNA gene are employed for accurate identification [5,6]. For example, identifying *Salmonella* Typhimurium from other *Salmonella enterica* serovars using phenotypic or chemical properties can be challenging due to their similarities in biochemical characteristics [7]. Although serotyping is highly effective in distinguishing between the two sub-species of *Salmonella*, it is not routinely done [8]. In addition, some commercial systems do not have *S.* Typhimurium or misdiagnose it, leading to under-recognition [7].

There are several genotypic methods for the detection of AMR determinants in bacteria [9]. These genotypic methods are essential tools in microbiology, helping to inform treatment decisions, track resistance trends, and understand the genetic mechanisms underlying antimicrobial resistance. The choice of method often depends on the specific context, including the required sensitivity, specificity, speed, and resources available [10]. Multiplex PCR assays that amplify specific AMR determinants have high sensitivity and specificity and can detect multiple genes simultaneously [11]. In Kenya, most hospitals use GeneXpert [12] for detection of mycobacterium AMR determinants [13]. But the test has broader capability, including detection of carbapenemases [12] and *vanA/vanB* [14]. Of the molecular methods, next generation whole genome sequencing (WGS) is the most robust, has high-throughput capability that allows for the simultaneous detection of multiple genes or entire genomes, thus can provide comprehensive data on AMR determinants, including novel mutations and plasmid content [10,15]. Using bioinformatics tools and reference databases, WGS is able to accurately identify bacteria, determine the AMR determinants they carry, including resistance mechanisms [16,17]. This study compared phenotypic (AST) and WGS methods in bacterial species identification and assessment of AMR determinants in blood cultures obtained from children who had antibiotic prescription at admission.

## Materials and methods

### Study site, ethical approval and subject enrollment

The study was part of a multi-laboratory AMR project that included KEMRI Centre for Microbiology Research for sites in Nairobi and Central Kenya, KEMRI Wellcome Trust Research Programme for sites in the Coastal region and KEMRI Walter Reed Army Institute of Research-Africa (WRAIR-A) for sites in Nyanza and Western Kenya. Details of the study protocol has been published before [18]. The inclusion criteria was all children where antibiotic prescription was made at admission. Such children were eligible for blood culture. For the KEMRI/WRAIR-A sites, the study was conducted at the Kombewa Clinical Research Center (CCR), Kisumu Field Station, where active recruitment commenced on 7^th^ March 2022 and ended on 30^th^ November 2023 and collected samples from county referral hospitals in western Kenya and around the Lake Victoria region (Busia, Kakamega, Homabay, Kisumu and Vihiga). Children aged 1 month to 12 years, to whom antibiotics had been prescribed upon admission to pediatric wards were recruited into the study. The study was approved by the Kenya Medical Research Institute Scientific and Ethics Review Unit (KEMRI SERU, # 4246) and the WRAIR Human Subject Protection Branch, (WRAIR #2888). Approval for waiver of consent was obtained from SERU on August 10, 2021 as blood cultures are deemed routine and the microbiology results from the study are used for clinical management of individual patients.

### Culture and phenotypic antimicrobial susceptibility testing

960 blood cultures (BCs) were collected and incubated in BD Bactec 9050 (Sparks, Maryland, USA). BCs with growth were sub-cultured on blood agar, chocolate blood agar, and MacConkey agar. Following Gram staining, the bacteria identification and AST profiles were performed using the BD Phoenix 100 system with software v6.01 and Phoenix Update Disk (PUD) v5.11. The bloodstream bacteria of interest were those in the World Health Organization - Global antimicrobial resistance and use surveillance (WHO-GLASS) priority list, namely *Escherichia coli, Klebsiella pneumoniae, Acinetobacter baumannii, Staphylococcus aureus, Streptococcus pneumoniae, Salmonella* spp. [19]. All the other bacteria were considered not clinically relevant or contaminants and were dropped from further consideration.

### Whole genome sequencing

BCs for sequencing were subcultured for purity in 5% Columbia blood agar (Thermo Fisher Diagnostics) and incubated overnight at 37°C. A colony was picked with a loop and added to 250 μL of phosphate buffered saline. Genomic DNA was extracted using the ZymoBIOMICS DNA/RNA Miniprep Kit (Zymo RESEARCH, California, US), according to the manufacturer’s instructions. Genomic DNA library was prepared for sequencing using the Native Barcoding Kit 24 v14 (SQK-NBD114.24) (Oxford Nanopore Technologies, Oxford, UK), according to the manufacturer’s instructions. 200 μL of the prepared DNA library was loaded into an R10.4.1 flow cell and then sequenced on the PromethION 2 Solo instrument (Oxford Nanopore Technologies, Oxford, UK) for a maximum of 72 hours. Raw pod5 files from the PromethION were base called using Dorado v0.5.2 (https://github.com/nanoporetech/dorado), with the super accurate (SUP) basecalling mode. The demultiplexed sequences were then run on EDGE Bioinformatics v2.4.0 Pipeline [20], that incorporates quality control using FaQCs v1.34 [21]; assembly using Flye v.2.9.3 [22]; and taxonomic classification using Kraken v1.2.0 [23] to identify the bacteria. For annotation, the Flye assembled contigs were further analyzed for AMR determinants using abritAMR v1.0.13 [24], with the NCBI AMRFinderPlus database [25], retaining only core AMR genes, and with the Resistance Gene Identifier (RGI) v6.0.3 (https://card.mcmaster.ca/analyze/rgi) against the Comprehensive Antimicrobial Resistance Database (CARD) v3.2. [26]. The abritAMR’s ≥ 90% reference sequence identity and coverage threshold were used in both tools (23). An isolate was defined as genotypic resistant by the presence of at least one AMR gene (ARG) or chromosomal point mutation (PM) known to confer resistance to a given antimicrobial agent and as genotypic susceptible when noARG or PM was found [27,28].

### Confirmation of the identity of *Salmonella* isolates by phylogenetic analysis

To confirm the identity of the *Salmonella* isolates, phylogenetic analysis was done with both *S.* Typhimurium and *S.* Typhi global sequences downloaded from the NCBI database (https://blast.ncbi.nlm.nih.gov). We supplemented the NCBI Gen bank sequences with high-quality African isolates from Enterobase database v1.2.0 (https://enterobase.warwick.ac.uk) [29]. For *S.* Typhimurium sequences, we performed a systematic search in NCBI GenBank using the query “Salmonella enterica serovar Typhimurium[Organism] AND complete genome[Title]”. To ensure geographic and temporal diversity, we stratified available genomes by Geographic origin (Africa: n = 19, Asia: n = 11, Europe: n = 3, Americas: n = 4, Oceania: n = 3), isolation year (1911–2000: n = 2, 2000–2005: n = 8, 2006–2010: n = 7, 2011–2015: n = 8, 2016–2020: n = 7, 2021-present: n = 8). Random selection was performed within each stratum to avoid selection bias. These sequences were supplemented with high-quality African isolates from EnteroBase to ensure regional representation relevant to our study context. For the *S*. Typhi sequences, similar stratified sampling approach was used. We prioritized complete genomes over draft assemblies where available, geographic distribution: Africa: n = 13, Asia: n = 9, Europe: n = 3, Americas: n = 4, Oceania: n = 1). The following quality filters were used: assembly level = complete chromosome or scaffold, N50 > 1Mb, and recent annotation.

The core-genome maximum likelihood phylogenetic tree was created using Parsnp v2.0.3 [30], employing the generalized time-reversible (GTR) with CAT rate heterogeneity model, 1000 bootstrap replicates (values >70% considered well-supported) [31]. The created tree was visualized in iTOL v6.9 with metadata mapping [32]. Manual inspection was performed to ensure that sequences producing unusually long tree branches are truly representative and not artifacts of misassembly, misalignment, or lab contamination.

## Results

### Blood cultures failed to identify *Salmonella* Typhimurium

Out of the 960 blood cultures, 123 (12.8%) had bacterial growth, and of these, only 17 (13.8%) were usable, as determined by the Global Antimicrobial Resistance and use Surveillance System (GLASS) priority list of pathogens [19]. Metadata of the study isolates are shown in Supplementary information (S1 Table). Phenotypically, the bacteria were classified as 4 Gram positives: *S. aureus* (n = 3), and *S. pneumoniae* (n = 1) and 13 Gram negatives: *E. coli* (n = 4), *S.* Typhi*,* (n = 8), and unspeciated *Salmonella* (n = 1). WGS was concordant with phenotypic identification of all other bacteria, apart from the 9 *Salmonella* isolates. By WGS, the *Salmonella* isolates were identified as *Salmonella enterica* serovar Typhimurium (S1 Table).

**Fig 1:**
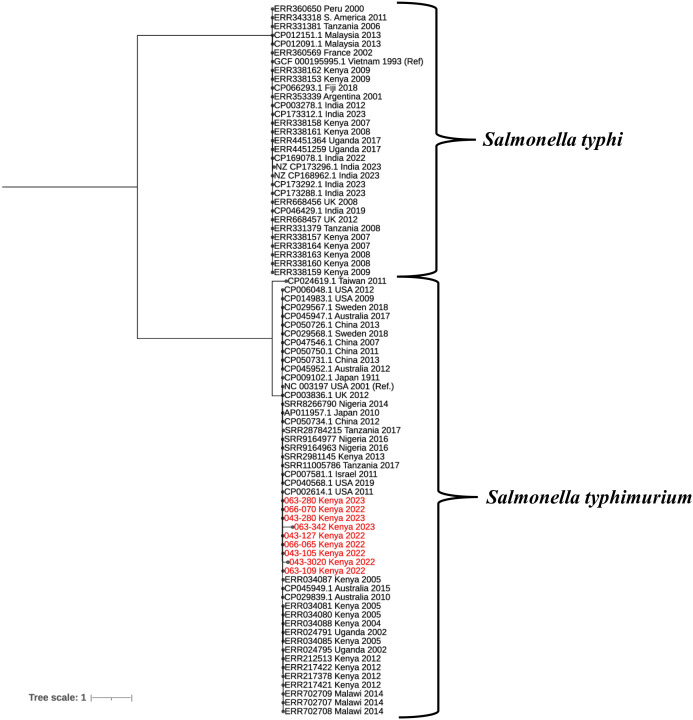
A maximum Likelihood Phylogenetic tree generated from *Salmonella* core genome single nucleotide polymorphisms using Parsnp v2.0.3. The study genomes (highlighted in red) clustered with *S.* Typhimurium and not *S.* Typhi. GCF_0001959951 = *S*. Typhi reference genome; NC_003197 = *S.* Typhimurium reference genome.

S3 Table details accession numbers from public databases that were used for phylogenetic tree construction. By phylogenetic analysis, the *Salmonella* study genomes formed a single cluster with the global *Salmonella enterica* serovar Typhimurium sequences (Fig 1, red fonts). The genome sequences of all the 17 pathogenic bacteria have been deposited in GenBank under bioproject number PRJNA1254897.

### Antimicrobial resistance profiles

Detailed phenotypic resistance profiles for *Salmonella* spp*., E. coli*, *S. aureus* and *S. pneumoniae* are shown in Table 1.

**Table 1:** Antibiotic resistance profile for *Salmonella* spp*., Escherichia coli*, *Staphylococcus aureus* and *Streptococcus pneumoniae* were performed in the BD Phoenix 100 system.

**Gram-positive Bacteria**			
**Antibiotic** **Class**	**Antibiotics Tested**	**Resistance (%)**	
		***S. aureus*, n = 3**	***S. pneumoniae,* n = 1**
Sulfonamides	Trimethoprim-Sulfamethoxazole	100	0
Beta-lactams	Ampicillin	100	NT
	Penicillin	100	NT
	Ceftaroline	0	NT
	Cefepime	NT	0
	Oxacillin	0	NT
	Cefotaxime	NT	0
Tetracycline	Tetracycline	33.3	0
Macrolide	Clindamycin	0	0
	Erythromycin	0	0
Lipopeptide	Daptomycin	0	NT
Oxazolidinone	Linezolid	0	NT
Glycylcyclines	Tigecycline	0	NT
Rifamycin	Rifampin	0	NT
Glycopeptide	Vancomycin	0	0
Quinolones	Levofloxacin	NT	0
	Moxifloxacin	NT	0
	Meropenem	NT	0
**Gram-Negative Bacteria**			
**Antibiotic** **Class**	**Antibiotics** **Tested**	**Resistance (%)**	
		***Salmonella*,** **n = 9**	***E. coli*, n = 4**
Aminoglycosides	Amikacin	100	0
	Gentamicin	100	25
Sulfonamides	Trimethoprim-Sulfamethoxazole	88.9	50
Beta-lactams	Ampicillin	88.9	50
	Cefazolin	100	50
	Cefuroxime	100	50
	Ceftazidime	0	25
	Ceftolozane-Tazobactam	0	25
	Ceftriaxone	0	50
	Amoxicillin-Clavulanate	0	0
	Cefepime	0	25
	Ertapenem	0	25
	Imipenem	0	0
	Meropenem	0	25
	Piperacillin-Tazobactam	0	0
Glycylcyclines	Tigecycline	0	0
Quinolones	Ciprofloxacin	ND	25
	Levofloxacin	ND	25

NT = Not Tested by the BD Phoenix 100; ND = Not determined by the BD Phoenix 100.

**Fig 2:**
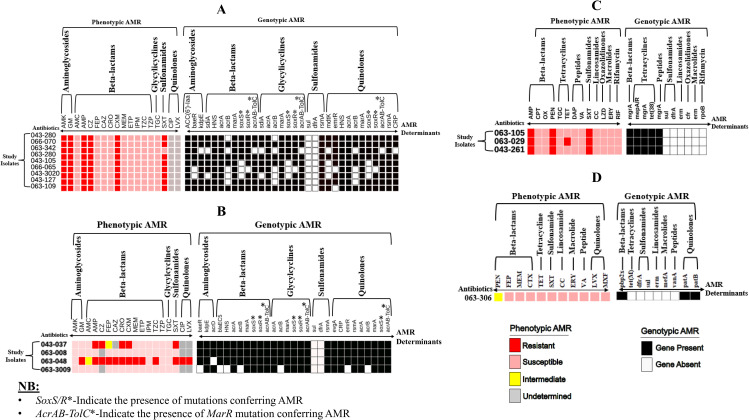
Phenotypic and genotypic AMR profiles of the study isolates: *Salmonella* spp. (Panel A), *Escherichia coli* (Panel B), *Staphyloccucus aureus* (Panel C), and *Streptococcus pneumoniae* (Panel D) to the following antibiotics. Aminogylcosides (Amikacin = AMK, Gentamicin = GM); Beta-lactams (Amoxicillin-Clavulanate = AMC, Ampicillin = AMP, Cefazolin = CZ, Ceftazidime = CAZ, Ceftriaxone = CRO, Cefuroxime = CXM, Meropenem = MEM, Ertapenem = ETP, Imipenem = IPM, Ceftolozane-Tazobactam = TZC, Ceftaroline = CPT, Oxacillin = OX, TZP = Piperacillin-Tazobactam); Glycylcycline (Tigecycline = TGC); Sulfonamides (Trimethoprim-Sulfamethoxazole = SXT) Quinolones (Ciprofloxacin = CIP, Levofloxacin = LVX).

Fig 2 shows the phenotypic and genotypic AMR for the study isolates. All the nine isolates of *Salmonella* (Panel A) showed 100% resistance to aminoglycosides (amikacin and gentamycin) and sulfonamide (trimethoprim-sulfamethoxazole). The isolates had 100% sensitivity to beta-lactams, except ampicillin, cefazolin, and cefuroxime that had 88.9%, 100% and 100% resistance respectively and 100% sensitivity to glycylcycline (tigecycline). There was a higher phenotype and genotype concordance in the resistance profile to aminoglycosides. AMR determinants that code for beta-lactams resistance were identified in both the susceptible and resistant isolates. Despite the *Salmonella* isolates being susceptible to tigecycline, multiple AMR determinants were identified. For sulfonamides, only the ***rsm****A* resitance gene was present in all the *Salmonella* isolates.

In all instances except one, the *E. coli* isolates (Fig 2, Panel B) were 100% sensitive to aminoglycosides, but had variable resistance to beta-lactams, sulfonamide and quinolones and 100% sensitivity to glycylcycline (tigecycline). Although *E. coli* isolates showed high sensitivity to aminoglycosides, multiple AMR determinants that code for resistance to amonoglycosides were identified. Likewise, AMR determinants that code for beta-lactams resistance were identified in both the susceptible and resistant *E. coli*. Similar to the *Salmonella* isolates, multiple AMR determinants that code for resistance to tigecycline were identified despite the susceptible phenotype. For sulfonamides, only the *rsmA* resistance gene was present in all the *E. coli* isolates. AMR determinants that confer resistance to quinolones were present in nearly all the *E. coli* isolates.

By culture and sensitivity, the *S. aureus* isolates (Fig 2, Panel C) were completely resistant to two beta- lactams (ampicillin and penicillin) and sulfonamide. One isolate was resistant to tetracycline. The isolates were completely susceptible to the other antibiotics tested (ceftaroline, oxacillin tigecycline, daptomycin, vancomycin, linezolid, clindamycin, erythromycin and rifamycin). The AMR presence/absence of genes did not match the expected phenotype profile. Unlike the *Salmonella* isolates, it was the *LmrS* gene that codes for sulfonamide resistance that was identified in all the *S. aureus* isolates, and not *rsmA* gene.

The phenotypic and genotypic profile of the lone isolate of *S. pneumoniae* is shown in Fig 2 (Panel D). The isolate was susceptible to all the tested antibiotics. AMR determinants coding for resistance to beta-lactams and quinolones were identified, despite the isolates being sensitive to these antibiotics. In concordance to AST profile, AMR determinants coding for resistance to tetrcyclines, sulfonamides, lincosamide, macrolide and peptides were absent.

## Discussion

Bacterial infections in the bloodstream are a major cause of sepsis and fatalities, making precise identification of the causative agents and their AMR profiles essential for effective management [3,33]. In this study, blood for BC was collected at admission, prior to antibiotic initiation, from children admitted to participating hospitals in western Kenya. Clearly, not all such children needing antibiotic initiation had blood stream bacteremia. This may in part explain the low recovery of pathogenic bacteria, 17 out of 960, following BC. Even in cases of septicemia, low recovery from BC is common [34–36]. Factors such as presence of fastidious or uncultivable organisms, prior antimicrobial use, or limitations in the culture techniques are blamed. Secondary, the bloodstream bacteria of interest were the six in the WHO-GLASS antimicrobial resistance and use surveillance priority list, namely *Escherichia coli*, *Klebsiella pneumoniae*, *Acinetobacter baumannii*, *Staphylococcus aureus*, *Streptococcus pneumoniae*, and *Salmonella spp*. All the other bacteria were dropped from further analysis.

Phenotypic and genotypic markers by AST and WGS respectively were largely concordant in identification of the bacterial isolates, except for the assignment of *Salmonella* serovars. Phenotypic markers identified them as either *Salmonella* spp. or *S*. Typhi, while genotypic markers identified them as *S.* Typhimurium. *Salmonella enterica* serovars have similar biochemical characteristic that make it difficult to distinguish them [7]. Multiple other studies have found WGS to have better discriminative power compared to phenotypic methods [8,37,38]. Two, as shown in Fig 1, the 9 *Salmonella* study genomes clustered with the global *S.* Typhimurium, and not *S.* Typhi. Three, we noted that the BD Phoenix 100 bacterial ID list [38] has *S.* Typhi but no *S.* Typhimurium. Misclassification of *S.* Typhimurium as *S.* Typhi can lead to underestimation of the burden of *S.* Typhimurium. Correct classification is also important because *Salmonella enterica* serovars have distinct clinical presentations, epidemiology, and treatment requirements [39]. In humans, *S.* Typhimurium typically causes gastroenteritis, characterized by diarrhea, abdominal pain, fever, and vomiting. In severe cases, it can lead to systemic infection, particularly in immunocompromised individuals. [40,41]. In contrast, *S.* Typhi causes a systemic infection affecting multiple organs [42].

As illustrated in Fig 2 (Panel A), *S.* Typhimurium showed 100% sensitivity to beta-lactams, apart from ampicillin, cefazolin, and cefuroxime. Previous studies in Kenya had showed non-sensitivity to most beta-lactams [43–45]. AMR determinants that code for beta-lactam resistance were identified in both the susceptible and resistant isolates. Unlike the beta-lactams, there was a better phenotype and genotype concordance in the resistance profile to aminoglycosides (amikacin and gentamicin). Similar to our study, several others have found trimethoprim-sulfamethoxazole resistance in *S.* Typhimurium which was attributed to *sul* and *dfrA* AMR genes [45–47]. In this work, we only identified the *rsmA* gene that is known to confer resistance to trimethoprim-sulfamethoxazole. Similarly, AMR determinants that code for glycylicyclines (tigecycline) were identified despite all *S.* Typhimurium isolates being phenotypically susceptible.

*E. coli*, which mostly resides in the gut of humans and animals, is mainly transmitted through consumption of contaminated food and water, and has the ability to survive and reproduce in the environment [48]. As shown in Fig 2 (Panel B), *E. coli* isolates were phenotypically sensitive to aminoglycosides (amikacin and gentamicin), suggesting their potential as effective treatment options. The observed phenotypic resistance to beta-lactams, sulfonamides (e.g., trimethoprim-sulfamethoxazole), and quinolones aligns with the widespread prevalence of AMR determinants for these drug classes. Similar to *S.* Typhimurium, the observed presence of the *rsmA* resistance gene across all gram negative isolates may explain the observed resistance to sulfonamides. This gene likely plays a key role in resistance to this class of antibiotics. The phenotypic susceptibility to tigecycline is promising, suggesting its potential as a robust treatment option for *E. coli* infections. However, the detection of multiple AMR determinants associated with tigecycline resistance is concerning.

The phenotypic and genotype profiles of the three isolates of *S. aureus* are shown in Fig 2 (Panel C). The isolates were completely resistant to ampicillin and penicillin, but susceptible to ceftaroline and oxacillin. The isolates were also resistant to trimethoprim-sulfamethoxazole and had variable resistance to tetracycline. The phenotypic resistance to beta-lactams (ampicillin and penicillin), sulfonamides (trimethoprim-sulfamethoxazole) and tetracycline is similar to previous studies done in Kenya [49–52]. Irrespective of phenotypic resistance profiles, AMR determinants were identified in all isolates apart from lincosamide and rifamycin. *S. pneumoniae* was susceptible to all the tested antibiotics (Fig 2, Panel D). Resistance genes were only found for beta-lactams, and quinolones, and none for tetracyclines, sulfonamides, lacosamides, macrolides and peptides. In general, the phenotype profile agrees with a systematic review by Droz et al that found high susceptibility of *S. pneumoniae* to beta-lactams in Africa [53]. In another study done in Kenya [54], *S. pneumoniae* was found to have increased resistance to oxacillin and erythromycin, which is contrary to our findings. Lastly, our study had only one isolate of *S. pneumoniae* and hence making it difficult to generalize these findings.

Despite the enormous potential of WGS in diagnosis, AMR detection, among others, its uptake as a routine clinical tool has been slow. Among the many reasons for the slow uptake include the long workflow for WGS (DNA extraction, sequencing, analysis, interpretation often take 24–72 hours) which is too long, costly and complex for clinicians' need at bedside. Other reasons include regulatory and quality control gaps. Nevertheless, there are several studies that have concluded that WGS genotyping provides as reliable results as those of phenotypic AST. For example, Bortolaia et al [55], Rebelo et al [27], Schwan et al [28], found >90% genotype/phenotype concordance.

An even complex scenario is the role of WGS-based AMR detection in guiding therapy decisions. Today, current guidance stresses that it should complement rather than replace phenotypic AMR in most clinical contexts. The gap between genotype and phenotype, especially in Gram-negative bacteria with complex, polygenic resistance mechanisms, limits the reliability of WGS for direct therapeutic decision-making [56,57]. An important exception is *Mycobacterium tuberculosis*, where a curated WHO mutation catalogue allows WGS to reliably predict resistance and guide therapy [58].

In conclusion, phenotypic markers failed to correctly identify the serovars of *Salmonella enterica* and classified them as *Salmonella* spp*.* or *S.* Typhi*.* WGS correctly identified the misclassified *Salmonella* as *S.* Typhimurium. Additionally, our results highlight the importance of integrating genotypic and phenotypic data for a comprehensive understanding of AMR. Phenotypic sensitivity testing alone may underestimate the potential for resistance development, especially when silent or partially expressed AMR determinants are present. Continued surveillance of AMR determinants, even in phenotypically susceptible isolates, is critical to predict and mitigate emerging resistance threats. These results underscore the need for combination therapies or alternative treatment approaches to address variable resistance profiles, especially for beta-lactams, sulfonamides, and quinolones. A limitation for the study is that the genotypic AMR was predicted at the antibiotic class level while phenotypic AST was at the individual antibiotic level. This makes it impossible to calculate positive/negative percentage agreements between the two methods. This limitation is inherent to the genotypic AMR detection tools used in this study. In addition, treatment and patient outcome data were not available, which limits the ability to evaluate the clinical significance of genotype/phenotype discrepancies observed in this study.

## Supporting information

S1 Table Metadata of study samples. (XLSX)

S2 Table Bacterial identity comparisons between BD Phoenix 100 and WGS. (XLSX)

S3 Table Metadata of global sequences. (XLSX)

## References

[1] Aljeldah MM. Antimicrobial Resistance and Its Spread Is a Global Threat. Antibiotics (Basel). 2022;11(8):1082. doi: 10.3390/antibiotics11081082 36009948 PMC9405321

[2] Godman B, Egwuenu A, Wesangula E, Schellack N, Kalungia AC, Tiroyakgosi C, et al. Tackling antimicrobial resistance across sub-Saharan Africa: current challenges and implications for the future. Expert Opin Drug Saf. 2022;21(8):1089–111. doi: 10.1080/14740338.2022.2106368 35876080

[3] Seni J, Mwakyoma AA, Mashuda F, Marando R, Ahmed M, DeVinney R, et al. Deciphering risk factors for blood stream infections, bacteria species and antimicrobial resistance profiles among children under five years of age in North-Western Tanzania: a multicentre study in a cascade of referral health care system. BMC Pediatr. 2019;19(1):32. doi: 10.1186/s12887-019-1411-0 30684964 PMC6347777

[4] McEwen SA, Collignon PJ. Antimicrobial Resistance: a One Health Perspective. Microbiol Spectr. 2018;6(2). doi: 10.1128/microbiolspec.ARBA-0009-2017 29600770 PMC11633550

[5] Yamin D, Uskoković V, Wakil AM, Goni MD, Shamsuddin SH, Mustafa FH, et al. Current and Future Technologies for the Detection of Antibiotic-Resistant Bacteria. Diagnostics (Basel). 2023;13(20):3246. doi: 10.3390/diagnostics13203246 37892067 PMC10606640

[6] Galluzzi L, Magnani M, Saunders N, Harms C, Bruce IJ. Current molecular techniques for the detection of microbial pathogens. Sci Prog. 2007;90(Pt 1):29–50. doi: 10.3184/003685007780440521 17455764 PMC10361161

[7] Awang MS, Bustami Y, Hamzah HH, Zambry NS, Najib MA, Khalid MF, et al. Advancement in Salmonella Detection Methods: From Conventional to Electrochemical-Based Sensing Detection. Biosensors (Basel). 2021;11(9):346. doi: 10.3390/bios11090346 34562936 PMC8468554

[8] Diep B, Barretto C, Portmann A-C, Fournier C, Karczmarek A, Voets G, et al. Salmonella Serotyping; Comparison of the Traditional Method to a Microarray-Based Method and an in silico Platform Using Whole Genome Sequencing Data. Front Microbiol. 2019;10:2554. doi: 10.3389/fmicb.2019.02554 31781065 PMC6859910

[9] van Belkum A, Dunne WM Jr. Next-generation antimicrobial susceptibility testing. J Clin Microbiol. 2013;51(7):2018–24. doi: 10.1128/JCM.00313-13 23486706 PMC3697721

[10] Salam MA, Al-Amin MY, Pawar JS, Akhter N, Lucy IB. Conventional methods and future trends in antimicrobial susceptibility testing. Saudi J Biol Sci. 2023;30(3):103582. doi: 10.1016/j.sjbs.2023.103582 36852413 PMC9958398

[11] Anjum MF, Zankari E, Hasman H. Molecular Methods for Detection of Antimicrobial Resistance. Microbiol Spectr. 2017;5(6):10.1128/microbiolspec.arba-0011–2017. doi: 10.1128/microbiolspec.ARBA-0011-2017 29219107 PMC11687549

[12] Ding L, Shi Q, Han R, et al. Comparison of four carbapenemase detection methods for bla KPC-2 variants. Microbiol Spectr. 2021;9:1–10. 10.1128/Spectrum.00954-21 PMC8693920 34935416

[13] Bwana P, Ageng’o J, Mwau M. Performance and usability of Cepheid GeneXpert HIV-1 qualitative and quantitative assay in Kenya. PLoS One. 2019;14(3):e0213865. doi: 10.1371/journal.pone.0213865 30901343 PMC6430374

[14] Li Z-L, Luo Q-B, Xiao S-S, Lin Z-H, Liu Y-L, Han M-Y, et al. Evaluation of GeneXpert vanA/vanB in the early diagnosis of vancomycin-resistant enterococci infection. PLoS Negl Trop Dis. 2021;15(11):e0009869. doi: 10.1371/journal.pntd.0009869 34748586 PMC8575182

[15] Tamma PD, Fan Y, Bergman Y, Pertea G, Kazmi AQ, Lewis S, et al. Applying Rapid Whole-Genome Sequencing To Predict Phenotypic Antimicrobial Susceptibility Testing Results among Carbapenem-Resistant Klebsiella pneumoniae Clinical Isolates. Antimicrob Agents Chemother. 2018;63(1):e01923-18. doi: 10.1128/AAC.01923-18 30373801 PMC6325187

[16] Sydenham TV, Overballe-Petersen S, Hasman H, Wexler H, Kemp M, Justesen US. Complete hybrid genome assembly of clinical multidrug-resistant Bacteroides fragilis isolates enables comprehensive identification of antimicrobial-resistance genes and plasmids. Microb Genom. 2019;5(11):e000312. doi: 10.1099/mgen.0.000312 31697231 PMC6927303

[17] Su M, Satola SW, Read TD. Genome-Based Prediction of Bacterial Antibiotic Resistance. J Clin Microbiol. 2019;57(3):e01405-18. doi: 10.1128/JCM.01405-18 30381421 PMC6425178

[18] Akech S, Nyamwaya B, Gachoki J, Ogero M, Kigo J, Maina M, et al. The CINAMR (Clinical Information Network-Antimicrobial Resistance) Project: A pilot microbial surveillance using hospitals linked to regional laboratories in Kenya: Study Protocol. Wellcome Open Res. 2022;7:256. doi: 10.12688/wellcomeopenres.18289.1 37786881 PMC10541537

[19] GLASS. Global Antimicrobial Resistance and Use Surveillance System (GLASS) Report 2021. 2021.

[20] Li P-E, Lo C-C, Anderson JJ, Davenport KW, Bishop-Lilly KA, Xu Y, et al. Enabling the democratization of the genomics revolution with a fully integrated web-based bioinformatics platform. Nucleic Acids Res. 2017;45(1):67–80. doi: 10.1093/nar/gkw1027 27899609 PMC5224473

[21] Lo C-C, Chain PSG. Rapid evaluation and quality control of next generation sequencing data with FaQCs. BMC Bioinformatics. 2014;15(1):366. doi: 10.1186/s12859-014-0366-2 25408143 PMC4246454

[22] Kolmogorov M, Yuan J, Lin Y, Pevzner PA. Assembly of long, error-prone reads using repeat graphs. Nat Biotechnol. 2019;37(5):540–6. doi: 10.1038/s41587-019-0072-8 30936562

[23] Wood DE, Salzberg SL. Kraken: ultrafast metagenomic sequence classification using exact alignments. Genome Biol. 2014;15(3):R46. doi: 10.1186/gb-2014-15-3-r46 24580807 PMC4053813

[24] Sherry NL, Horan KA, Ballard SA, Gonҫalves da Silva A, Gorrie CL, Schultz MB, et al. An ISO-certified genomics workflow for identification and surveillance of antimicrobial resistance. Nat Commun. 2023;14(1):60. doi: 10.1038/s41467-022-35713-4 36599823 PMC9813266

[25] Feldgarden M, Brover V, Fedorov B, Haft DH, Prasad AB, Klimke W. Curation of the AMRFinderPlus databases: applications, functionality and impact. Microb Genom. 2022;8(6):mgen000832. doi: 10.1099/mgen.0.000832 35675101 PMC9455714

[26] Alcock BP, Huynh W, Chalil R, et al. CARD 2023: expanded curation, support for machine learning, and resistome prediction at the Comprehensive Antibiotic Resistance Database. Nucleic Acids Res. 2023;51:D690–9. 10.1093/nar/gkac920 PMC9825576 36263822

[27] Rebelo AR, Bortolaia V, Leekitcharoenphon P, Hansen DS, Nielsen HL, Ellermann-Eriksen S, et al. One Day in Denmark: Comparison of Phenotypic and Genotypic Antimicrobial Susceptibility Testing in Bacterial Isolates From Clinical Settings. Front Microbiol. 2022;13:804627. doi: 10.3389/fmicb.2022.804627 35756053 PMC9226621

[28] Schwan CL, Lomonaco S, Bastos LM, Cook PW, Maher J, Trinetta V, et al. Genotypic and Phenotypic Characterization of Antimicrobial Resistance Profiles in Non-typhoidal Salmonella enterica Strains Isolated From Cambodian Informal Markets. Front Microbiol. 2021;12:711472. doi: 10.3389/fmicb.2021.711472 34603240 PMC8481621

[29] Zhou Z, Alikhan N-F, Mohamed K, Fan Y, Agama Study Group, Achtman M. The EnteroBase user’s guide, with case studies on Salmonella transmissions, Yersinia pestis phylogeny, and Escherichia core genomic diversity. Genome Res. 2020;30(1):138–52. doi: 10.1101/gr.251678.119 31809257 PMC6961584

[30] Kille B, Nute MG, Huang V, et al. Parsnp 2.0: scalable core-genome alignment for massive microbial datasets. Bioinformatics. 2024;40:0–4. 10.1093/bioinformatics/btae311 PMC11128092 38724243

[31] Treangen TJ, Ondov BD, Koren S, Phillippy AM. The Harvest suite for rapid core-genome alignment and visualization of thousands of intraspecific microbial genomes. Genome Biol. 2014;15(11):524. doi: 10.1186/s13059-014-0524-x 25410596 PMC4262987

[32] Letunic I, Bork P. Interactive Tree Of Life (iTOL) v5: an online tool for phylogenetic tree display and annotation. Nucleic Acids Res. 2021;49(W1):W293–6. doi: 10.1093/nar/gkab301 33885785 PMC8265157

[33] Yang S, Xu H, Sun J, et al. Shifting trends and age distribution of ESKAPEEc resistance in bloodstream infection, Southwest China, 2012-2017. Antimicrob Resist Infect Control. 2019;8:1–10. 30976388 10.1186/s13756-019-0499-1 PMC6441235

[34] Murless-Collins S, Kawaza K, Salim N, Molyneux EM, Chiume M, Aluvaala J, et al. Blood culture versus antibiotic use for neonatal inpatients in 61 hospitals implementing with the NEST360 Alliance in Kenya, Malawi, Nigeria, and Tanzania: a cross-sectional study. BMC Pediatr. 2023;23(Suppl 2):568. doi: 10.1186/s12887-023-04343-0 37968606 PMC10652421

[35] Kisame R, Najjemba R, van Griensven J, Kitutu FE, Takarinda K, Thekkur P, et al. Blood Culture Testing Outcomes among Non-Malarial Febrile Children at Antimicrobial Resistance Surveillance Sites in Uganda, 2017-2018. Trop Med Infect Dis. 2021;6(2):71. doi: 10.3390/tropicalmed6020071 34066602 PMC8167719

[36] Lochan H, Pillay V, Bamford C, Nuttall J, Eley B. Bloodstream infections at a tertiary level paediatric hospital in South Africa. BMC Infect Dis. 2017;17(1):750. doi: 10.1186/s12879-017-2862-2 29207958 PMC5718141

[37] Lin J-N, Lai C-H, Yang C-H, Huang Y-H, Lin H-F, Lin H-H. Comparison of four automated microbiology systems with 16S rRNA gene sequencing for identification of Chryseobacterium and Elizabethkingia species. Sci Rep. 2017;7(1):13824. doi: 10.1038/s41598-017-14244-9 29062009 PMC5653830

[38] Phoenix ^TM^ Automated Microbiology System User’s Manua l. 003342.

[39] Id CN, Id CV, Akoko J, et al. Salmonella identified in pigs in Kenya and Malawi reveals the potential for zoonotic transmission in emerging pork markets. 2020;1–16. 10.1371/journal.pntd.0008796 PMC7748489 33232324

[40] Fàbrega A, Vila J. Salmonella enterica serovar Typhimurium skills to succeed in the host: virulence and regulation. Clin Microbiol Rev. 2013;26(2):308–41. doi: 10.1128/CMR.00066-12 23554419 PMC3623383

[41] Heredia N, García S. Animals as sources of food-borne pathogens: A review. Anim Nutr. 2018;4(3):250–5. doi: 10.1016/j.aninu.2018.04.006 30175252 PMC6116329

[42] Dougan G, Baker S. Salmonella enterica serovar Typhi and the pathogenesis of typhoid fever. Annu Rev Microbiol. 2014;68:317–36. doi: 10.1146/annurev-micro-091313-103739 25208300

[43] Kariuki S, Mbae C, Van Puyvelde S, Onsare R, Kavai S, Wairimu C, et al. High relatedness of invasive multi-drug resistant non-typhoidal Salmonella genotypes among patients and asymptomatic carriers in endemic informal settlements in Kenya. PLoS Negl Trop Dis. 2020;14(8):e0008440. doi: 10.1371/journal.pntd.0008440 32745137 PMC7425985

[44] Kariuki S, Okoro C, Kiiru J, Njoroge S, Omuse G, Langridge G, et al. Ceftriaxone-resistant Salmonella enterica serotype typhimurium sequence type 313 from Kenyan patients is associated with the blaCTX-M-15 gene on a novel IncHI2 plasmid. Antimicrob Agents Chemother. 2015;59(6):3133–9. doi: 10.1128/AAC.00078-15 25779570 PMC4432211

[45] Sora GH, Gachara G, Ichinose Y, et al. Antimicrobial Resistance Patterns of Bacterial Septicaemia Infecting Infants in Mbita Subcounty, Western Region of Kenya. J Nat Sci Res. 2020;10:50–61.

[46] Zhan Z, Xu X, Gu Z, et al. Molecular epidemiology and antimicrobial resistance of invasive non-typhoidal salmonella in China, 2007–2016. Infect Drug Resist. 2019;12:2885–97. 31571942 10.2147/IDR.S210961 PMC6750164

[47] Kubicek-Sutherland JZ, Xie G, Shakya M, Dighe PK, Jacobs LL, Daligault H, et al. Comparative genomic and phenotypic characterization of invasive non-typhoidal Salmonella isolates from Siaya, Kenya. PLoS Negl Trop Dis. 2021;15(2):e0008991. doi: 10.1371/journal.pntd.0008991 33524010 PMC7877762

[48] Jang J, Hur H-G, Sadowsky MJ, Byappanahalli MN, Yan T, Ishii S. Environmental Escherichia coli: ecology and public health implications-a review. J Appl Microbiol. 2017;123(3):570–81. doi: 10.1111/jam.13468 28383815

[49] Kohli-Kochhar R, Omuse G, Revathi G. A ten-year review of neonatal bloodstream infections in a tertiary private hospital in Kenya. J Infect Dev Ctries. 2011;5(11):799–803. doi: 10.3855/jidc.1674 22112734

[50] Kibaba PW, Louis H, Kering KK, et al. Antimicrobal susceptibility pattern of methicillin resistant Staphylococcus aureus isolated from pediatric clinical samples at Webuye District Hospital. 2017;74:238–50.

[51] Lord J, Gikonyo A, Miwa A, Odoi A. Antimicrobial resistance among Enterobacteriaceae, Staphylococcus aureus, and Pseudomonas spp. isolates from clinical specimens from a hospital in Nairobi, Kenya. PeerJ. 2021;9:e11958. doi: 10.7717/peerj.11958 34557345 PMC8418212

[52] Obanda BA, Gibbons CL, Fèvre EM, Bebora L, Gitao G, Ogara W, et al. Multi-Drug Resistant Staphylococcus aureus Carriage in Abattoir Workers in Busia, Kenya. Antibiotics (Basel). 2022;11(12):1726. doi: 10.3390/antibiotics11121726 36551383 PMC9774130

[53] Droz N, Hsia Y, Ellis S, Dramowski A, Sharland M, Basmaci R. Bacterial pathogens and resistance causing community acquired paediatric bloodstream infections in low- and middle-income countries: a systematic review and meta-analysis. Antimicrob Resist Infect Control. 2019;8:207. doi: 10.1186/s13756-019-0673-5 31893041 PMC6937962

[54] Orucho VO, Nyangau MK. Antibiotic resistance of Streptococcus pneumonia serotypes in Kisii, Kenya Cyrus Orucho Ochoi Kisii Teaching and referral hospital. 2020;1–10.

[55] Bortolaia V, Kaas RS, Ruppe E, Roberts MC, Schwarz S, Cattoir V, et al. ResFinder 4.0 for predictions of phenotypes from genotypes. J Antimicrob Chemother. 2020;75(12):3491–500. doi: 10.1093/jac/dkaa345 32780112 PMC7662176

[56] Ellington MJ, Ekelund O, Aarestrup FM, Canton R, Doumith M, Giske C, et al. The role of whole genome sequencing in antimicrobial susceptibility testing of bacteria: report from the EUCAST Subcommittee. Clin Microbiol Infect. 2017;23(1):2–22. doi: 10.1016/j.cmi.2016.11.012 27890457

[57] WHO. GLASS Whole-Genome Sequencing for Surveillance of Antimicrobial Resistance. 2020.

[58] World Health Organization. Catalogue of mutations in Mycobacterium tuberculosis complex and their association with drug resistance. 2021. Available from: https://www.who.int/publications/i/item/9789240028173

